# Foreign Object Debris Automatic Target Detection for Millimeter-Wave Surveillance Radar

**DOI:** 10.3390/s21113853

**Published:** 2021-06-02

**Authors:** Fei Qin, Xiangxi Bu, Yunlong Liu, Xingdong Liang, Jihao Xin

**Affiliations:** 1 National Key Lab of Microwave Imaging Technology, Aerospace Information Research Institute, Chinese Academy of Sciences, Beijing 100190, China; qinfei17@mails.ucas.edu.cn (F.Q.); buxx@aircas.ac.cn (X.B.); liuyunlong_cau@163.com (Y.L.); xinjihao19@mails.ucas.ac.cn (J.X.); 2 School of Electronics, Electrical and Communication Engineering, University of Chinese Academy of Sciences, Beijing 100049, China

**Keywords:** FOD, automatic target detection, millimeter-wave radar, clutter modeling, clutter map CFAR, long-distance detection

## Abstract

Foreign Object Debris (FOD) refers to any foreign material on the airfield that may injure and threaten the aircraft and airport system. Due to the complex background on the airfield pavement and weak target echoes in long-distance monitoring, it is not easy to detect objects of various types and sizes. The existing FOD radar system’s detection method has a short effective range, and the detectable objects’ radar cross-section intensity is no less than −20 dBsm. In this paper, we propose an integrated FOD automatic target detection algorithm for millimeter-wave (MMW) surveillance radar to improve small target detection under long-range conditions of over 660 m. The signal form of FOD and a clutter model of ground clutter received by millimeter-wave radar are primarily utilized and established theoretically. The runway edge detection means that it is employed based on the in-continuity features as the runway region of interest during the automatic extraction step. Following the clutter map constant false alarm detection algorithm, we utilize a time-domain algorithm that functions as the vital detection processor. Moreover, an explicit definition of the FOD detection performance is developed in a characteristic quantitative way. This criterion involves an absolute reference value for all FOD radar systems. The well-designed FOD frequency-modulated continuous-wave MMW surveillance radar is utilized, and actual experiments are carried out in a real airport in Beijing, China. The results validate the proposed method’s effectiveness and the superior performance of FOD target detection in long-range situations.

## 1. Introduction

Foreign Object Debris (FOD) at an airport includes any objects found in an inappropriate location that may damage equipment or poses risks to aircraft or airfield personnel [1,2]. According to a conservative estimate, the annual global loss caused by FOD to the aviation industry is approximately USD 3 to 4 billion [3]. FOD has become the most common threat to aviation security, second only to the hazards posed by birds. Following the International Civil Aviation Organization’s recommendations, the explicit inspection of runways must be performed at least four times per day [4]. While this strategy is not efficient, with high time consumption and costs associated with the manual inspection, an automated FOD detection system could provide an inspection tool that is 100% effective in guaranteeing the security of all flights.

The Federal Aviation Administration (FAA) classifies FOD programs into four areas [5]: detection, removal, evaluation, and prevention. Detection is the most critical part, and advanced sensor systems are developed to detect even millimeter-sized targets on the airfield. Typical examples of FOD include aircraft parts, twisted metal strips, metallic cylinders, spheres, screws, golf balls, plastic products, and so on. The above systems mainly realize FOD detection through radars and optical–electronic combinations.

At present, in terms of FOD detection system resolution, there are four representative and mature detection systems utilized worldwide [6,7,8,9]. They are Tarsier by QinetiQ, a frequency modulation continuous-wave radar deployed at Southampton and Vancouver airport; FOD Finder by Trex, a millimeter-wave (MMW) radar with a video camera that covers a 180 m range with high-speed turnoffs, which is deployed at Boston airport in the United States; FODetect by Xsight, an MMW radar and optical NIR illumination sensor used in Israel; iFerret by Stretch, a camera that can perform image processing under clear weather conditions at airports in Singapore; iFerret, which uses a high-fixed-resolution camera for foreign object detection. The remainder all take MMW radars as the primary detection sensors. While optical equipment will be weakened in inclement weather, radars can provide all-time and all-weather inspection for runways. In comparison, MMW radar is applied with minimal disruption to airport operations and robustness to hazardous weather, even in drizzling rain or light snow conditiona. The FOD finder can only detect targets as short as 2.5 cm × 2.5 cm (diameter × height), and its detection probability is the highest among the three MMW radar systems. Therefore, effective small target detection under long-distance conditions is an ongoing challenge.

To solve the FOD detection problem, researchers have proposed several practical algorithms recently. The algorithms are based on different sensors, including video cameras, active FMCW radar, and wide-band MMW radar, which could achieve good results under different weather conditions. Researchers mainly focus on image processing and radar signal processing. Traditional image processors utilize statistical and texture features, which often include a de-nosing filter. Gabor, bilateral filters, and texture segmentation are performed [10]. The constitutional neural network (CNN) algorithm [11,12] consists of a region proposal network and spatial transformer network. It utilizes a CNN classifier that can distinguish between screws and stones, while its target images have an average coverage of 80×80 pixels. The multi-directional, multi-scale weighted morphology FOD image de-noising method [13] can be applied by adjusting the structuring element. However, images obtained at night or in inclement weather are not ideal due to the influence of brightness and aliasing. In long-range scenarios, the targets would occupy only a few pixels; this is an insufficient coverage size to achieve acceptable performance.

In radar signal processing methods, the constant false alarm rate (CFAR) detection methods and some classification methods for detection are commonly used as FOD detection processors [14,15]. The CFAR algorithm comprises two kinds of resolution: spatial-domain and time-domain management. The Cell Average (CA) method has good detection performance with a homogeneous background; the The Greatest Of (GO) method works better at the clutter edges; the The Smallest Of (SO) method is best suited under conditions involving the appearance of many adjacent targets. The time-domain representative method Clutter Map (CM) CFAR algorithm could estimate the clutter power through multiple self-iterations [16,17]. As for classifiers, researchers have proposed a support vector domain description (SVDD) classifier with particle swarm optimization to detect [18]. It aims to gain high performance in detecting targets as small as a 2 cm metal ball within 50 m range. Though it serves as an MMW radar system, its process relies little on the extraction features; thus, the first-order and second-order central moment are utilized in the paper. This kind of processing algorithm is supervised detection, and the dataset is often unbalanced because the positive samples of FOD are far fewer than the negative samples of background. Another similar work used a higher-order statistic and SVDD classifier to improve the detection of five golf balls within a 70 m range [19,20]. The second-order central moment and fourth-order cumulants are fed to the classifier, which helps to distinguish false alarms among the detection candidates. Typical FOD RCS measurements are necessary for the design and data processing in a millimeter-wave radar system [21,22,23,24,25].

The background clutter severely deteriorates the target echoes, and this in turn interferes with the FOD detection process. The clutter inevitably restricts the performance of ground-based surveillance radar. At a long inspection distance, the grazing angle of the radar line of sight trails off. The Tarsier developer suggests an optimal grazing angle of around 2 degrees [26]. There are several interrelated clutter analyses for FOD systems. Researchers took into consideration a low grazing angle scenario and long-distance conditions of up to 200 km [27]. Their model comprised Rayleigh distribution as the amplitude modeling and Gaussian distribution for the power spectrum density, whereas simulated data of only four types of terrain relief were included and the resolution range of the results was not high in this work. In addition to the statistical approach, there are also zero memory nonlinear methods, such as the Weibull distribution model, and spherically invariant random process methods, such as the K distribution model [28]. Although geometric relationships are considered, this study lacks actual data to verify its validity. Reference [29] presented a new method for measuring the full-polarization scattering intensity of different road surfaces using a millimeter-wave radar. It is suitable for scenes with large elevation angles, but it is not suitable for settings with a low grazing angle. The comparison of various road surfaces was carried out using backscatter coefficients.

The valid inspection of the detection system results in a settlement plan and simultaneous work efficiency. As for a video monitoring system relying only on optical image processing, the iFerret system can detect targets of a height no shorter than 4 cm and it is restricted in terms of the weather conditions. As for the outstanding MMW radar systems, they have the capacity to detect FOD with a minimum radar cross-section (RCS) of −20 dBsm and a different coverage range of less than 300 m. Only the Tarsier claims up to a range of 2 km [26] without quantitative detection details. The RCS parameter is also an essential factor to consider as a reference for an FOD detection system. The critical factor of the inspection range is determined by many factors, e.g., system SNR, antenna, target RCS, and signal processing parameters. Therefore, it represents a promising means by which to improve the capacity of low RCS targets in long-distance detection application scenarios.

In this paper, we achieved the automatic target detection of remote and small debris on airfield pavements for an FOD surveillance radar. The process flowchart is presented in Section 3. It comprises runway ROI extraction based on signal form analysis, background clutter map establishment in the time domain, observation data registration, and clutter map detection processing. The experiment was carried out in an actual civil airfield runway in Beijing, China. Then, we measured the methods’ performance, and our designed FOD MMW surveillance radar presented an inspection radius of over 660 m of the runway distance. Rather than performing qualitative evaluation of the results, we assessed the performance with our explicitly defined evaluation indicators. It was validated that the method performs well to detect golf balls in long-range monitoring. The comparison results also demonstrated the superiority and explicitness of the proposed method.

A preliminary work of this paper was presented in [30,31]. The highlights and potential extensions of this paper can be summarized as follows.

1. An end-to-end automatic FOD detection resolution for the MMW surveillance radar is developed. It aims to resolve the difficulty of long-distance detection for smal-sized and typical low RCS targets such as golf balls.
2. The runway region of interest (ROI) extraction algorithm is employed based on the edge features of the background clutter model. The ROI zone can improve the efficiency and concentrate on the meaningful detection area.
3. The time-domain detection constant false alarm ratio processor is utilized and it performs better in terms of its higher detection probability, lower false alarm probability, and robustness of the airfield scenario.
4. An explicit criterion definition is demonstrated to evaluate the performance of the FOD detection system. Together with actual experiments in a real airport in Beijing, the proposed method is evaluated and discussed in detail.

The rest of this paper contains six main sections. In Section 2, the overall FMCW signal form and clutter modeling are given in detail, providing information for further automatic target detection. In Section 3, the runway ROI auto-extraction algorithm is employed based on in-continuity features of the clutter model, and the clutter map CFAR detection processor is utilized on account of the FMCW signal form of FOD targets. Section 4 further analyzes the experimental results and discusses the performance evaluation of our method. Section 5 provides a discussion of different target detection methods and their features regarding the defined criterion. Lastly, in Section 6, we briefly conclude this paper and provide some research directions for our future work.

## 2. FMCW Signal Form and Clutter Modeling

The FMCW scheme and clutter modeling aimed at runway and grasslands are utilized for millimeter-wave FOD surveillance radar systems. While the advantages of the FMCW application include its lower peak power compared to the pulse radar, the reduced costs, and low-profile hardware. Moreover, millimeter-wave radar has a large resolution range that reaches to centimeters and high sensitivity to targets. Considering the FOD system’s illumination geometry and scattering coefficient, we established a complex envelope of the airfield clutter model.

### 2.1. Signal Form of FMCW Radar

In every sweep cycle, the FMCW signal emitted by the FOD detection radar is performed as:

(1) $$s\left(t\right)=A\exp\left[j2\pi \left(f_{c}t+\frac{1}{2}ut^{2}\right)\right]$$

where *A* is the envelope form, $f_{c}$ is the carrier frequency, $u=B_{r}/T_{r}$ is the frequency modulation ratio, also known as the sweeping slope. $B_{r}$ is the bandwidth of the continuous wave, and $T_{r}$ is the duration of a single frequency sweep. For a stationary target on the airfield runway, the received echo signal of radar after the target reflection can be expressed as:

(2) $$s_{r}\left(t\right)=K_{\sigma }A\exp\left\{j2\pi \left[\left(f_{c}\left(t-\tau \right)+\frac{1}{2}u{\left(t-\tau \right)}^{2}\right)\right]\right\}$$

where $K_{\sigma }$ is the reflection coefficient related to the target RCS and τ=2R/c is the two-way time delay for a certain FOD target located in the range *R*. Submitting τ into the above Equation (2), the form is revised as follows:

(3) $$s_{r}\left(t\right)=K_{\sigma }A\exp\left\{j2\pi \left[\left(f_{c}t+\frac{1}{2}ut^{2}\right)\right]\right\}\exp\left[-j2\pi \left(u\frac{2R}{c}t-u\frac{2R^{2}}{c^{2}}+\frac{2R}{\lambda }\right)\right]$$

The echo signal is processed by down-conversion with the transmit signal filtering process; then, the beat frequency form is performed as:

(4) $$s_{r}\left(t\right)=K_{\sigma }A^{2}\exp\left[-j2\pi \left(u\frac{2R}{c}t-u\frac{2R^{2}}{c^{2}}+\frac{2R}{\lambda }\right)\right]$$

Performing an *N* point fast Fourier transform (FFT) on Equation (4), the signal frequency is characterized as:

(5) $$\begin{array}{c} S_{r}\left(n\right)=K_{\sigma }A^{2}N\sin c\left(n-\frac{2uR}{c}\frac{N}{f_{s}}\right)\exp\left[-j\pi \frac{N-1}{N}\left(n-\frac{2uR}{cf_{s}}\frac{N}{N-1}\right)\right]\\ \exp\left[j2\pi \left(u\frac{2R^{2}}{c^{2}}-\frac{2R}{\lambda }\right)\right] \end{array}$$

where $f_{s}$ is the sample frequency, $sinc\left(x\right)\;=sin\left(\pi x\right)/\left(\pi x\right)$ and n=1,2,3,…,N−1, whereas the range of the FOD target can be estimated from the spectrum of the $S_{r}\left(n\right)$ signal form.

A single beat frequency denoting $f_{beat}=2uR/c$. Thus, the distance of each FOD can be obtained via $R=\left(\frac{cf_{beat}}{2u}\right)$. The returned echo also contains noise components and clutter components.

### 2.2. Ground Clutter Modeling

As in our previously published work [30], clutter modeling in the airfield runway scenario comprises the non-Rayleigh distributed amplitude of clutter and the Gaussian distribution as the power spectrum density. It is based on the backscattering coefficient spatial correlated model; the long-range detection condition leads to a low grazing angle and FOD millimeter-wave radar format. In detail, the considered surface features are grassland and regular runway. We utilize Weibull distribution as the runway clutter amplitude and log-normal distribution as the grassland clutter amplitude.

**Figure 1:**
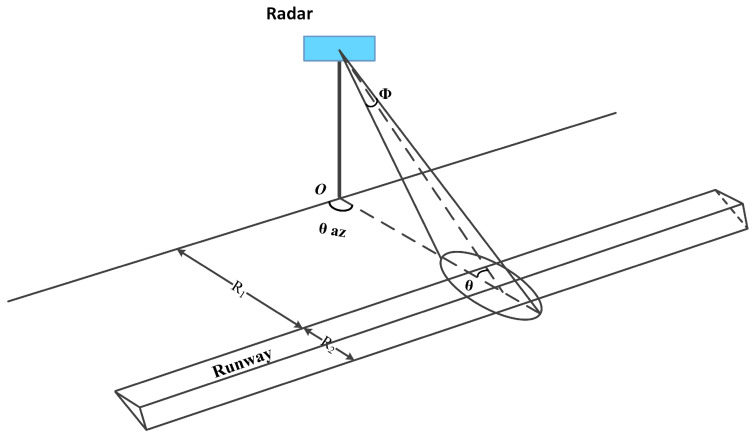
Geometry of FOD radar system.

There are two main types of FOD radar observation scenarios, namely airfield runway and nearby grass. Specifically, the grassland has non-homogeneous properties and the runway terrain is a quasi-smooth concrete surface. The surveillance radar system is expected to have a longer detection region, and the height of the equipment is strictly limited by the security rules. These combined factors lead to a small grazing angle when processing long-range detection conditions. It is vital to take into account the clutter scattering density and concise geometry relations. As shown in Figure 1, $R_{1}$ is the distance from the near edge of the runway to the radar’s location, $R_{2}$ is the breadth of the airfield pavement, $\theta_{az}$ denotes the azimuth scanning angle, ϕ represents the pitching beamwidth, and θ expresses the grazing angle.

Considering the relationship between the radar antenna pattern and the clutter distribution scenario, the radar backscattering intensity in every two-dimensional resolution unit is defined as:

(6) $$\sigma =\sigma_{0}A_{s}$$

The intensity comprises the normalized backscatter coefficient and two-dimensional resolution cell. To go one step further, the resolution cell can be divided into the wave beamwidth part, determined by the antenna pattern, and the slant range part. Projected onto the plane of the ground moment, the basic clutter resolution cell is demonstrated as:

(7) $$A_{s}=\Delta R\sec\left(\theta \right)R\theta_{\mathrm{az}}=\frac{c}{2B}\sec\left(\theta \right)R\theta_{az}$$

where $\theta_{az}$ denotes the azimuth beamwidth related to the antenna pattern. B represents the bandwidth of the signal form. θ expresses the grazing angle, which also equals the supplementary angle of the incident angle of the radar irradiation direction.

The received signal intensity is determined by the backscattering characteristics of different scenes and surface types—refer to the backscatter coefficient model proposed by Kulemin [32]. Through collecting a large amount of experimental data, this model is appropriate for the frequency range of 3–100 GHz and a grazing angle of no more than 30 deg. As for different pavement surfaces, it can be applied to several common types, including rough with and without vegetation, quasi-smooth concrete, and city areas. The backscatter coefficient is expressed in the form:

(8) $$\sigma_{0}=A_{1}+A_{2}\log\varphi /20+A_{3}\log f/10$$

where f denotes the carrier frequency in GHz form. φ is the grazing angle in degree form. The values of the $A_{1}-A_{3}$ coefficients for various surfaces are given in [30,32].

Modeling for complex scattering zones, we consider the Weibull and log-normal distribution as the clutter amplitude model. Existing test data have proven that the amplitude of ground clutter does not take the form of Gaussian or Rayleigh distribution [33]. Therefore, the long-tailed distribution is preferred to be used when describing the clutter amplitude. With consideration of the fact that the grassland fluctuations are much more severe than those of runways, the Weibull distribution is applied for the runway surface amplitude form. The actual experimental data also could be utilized to adjust these two kinds of distribution parameters.

## 3. Automatic Target Detection Method

**Figure 2:**
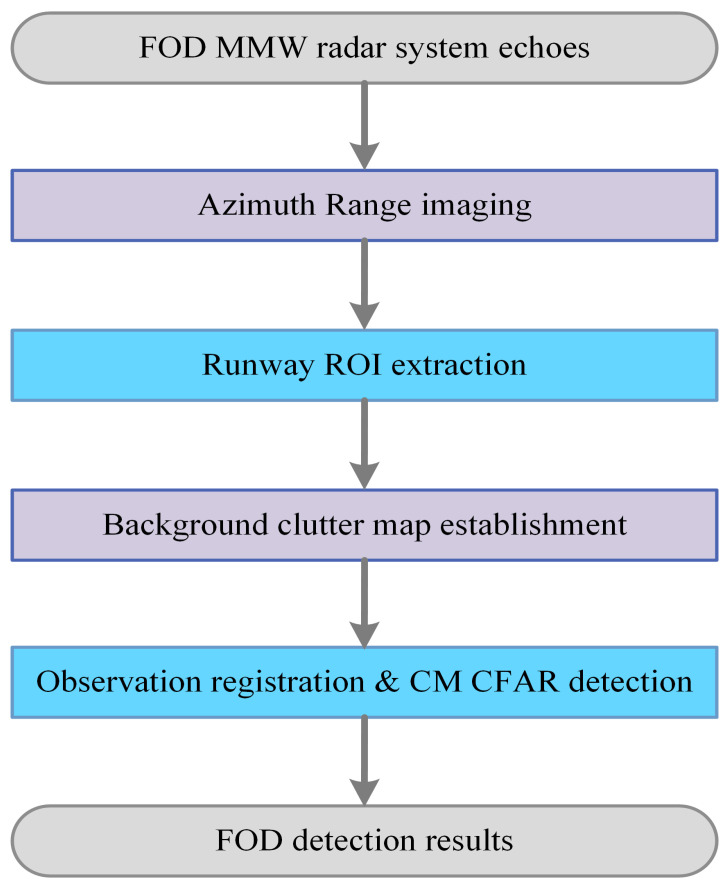
The processing flowchart of our automatic detection for FOD MMW radar.

With our proposed method, the clutter map was the critical detection processor. The flowchart of automatic FOD detection processing is shown in Figure 2. We adopt the point clutter map technique without spatial reference cells. We also realize the two-dimensional clutter map CFAR detection processor, but there are fewer gains and more false alarms in the clutter edges than in the one-dimensional case. The reason for this is the presence of small targets in the long-range scenarios.

### 3.1. Runway ROI Automatic Extraction

In order to decrease the unnecessary detection calculation and improve the efficiency, the runway ROI area should be extracted first. Typically, this is done by setting a fixed mask through as an interactive way to interpret the radar images. When the observation scenes change, they need to adjust again. The automatic ROI extraction algorithm will be illustrated next.

After match filtering of the beat frequency signal and servo scans of a full cycle, the two-dimensional azimuth-range images could be acquired—projected to the ground range according to the FOD system settlement geometry relation. In every inspection period, we utilize automatic ROI extraction processing. Taking into consideration the two kinds of terrain in the airfield, grassland and runway involve a different plural envelope distribution, which is analyzed in Section 2.2. Their backscatter amplitude varies in the edge between runway and grassland. Here, we utilize a bilateral filter for the de-noising of images and an edge detection algorithm based on the amplitude gradient information.

Bilateral filtering is a non-linear filter that incorporates a filtering model considering the amplitude value of neighboring pixels in Gaussian weight. It not only considers the Euclidean distance of pixels but also considers the radiation difference in the pixel range, which can achieve the effects of maintaining boundaries and smoothing noise. The formula for bilateral filtering is as follows:

(9) $$f\left(x,y\right)=\frac{\sum_{i,j\in S}w\left(i,j\right)g\left(i,j\right)}{\sum_{i,j\in S}w\left(i,j\right)}$$

where (i,j) is a two-dimensional index value, g(i,j) is the data before filtering, and *S* is the processing window.

As for the edge detection processing, we take the Canny second-order difference operator as a detector. The structural operators can be calculated in the horizontal and vertical directions. Then, we can obtain the gradient modulus $\sqrt{G_{x}^{2}+G_{y}^{2}}$ and angle $\theta =\arctan\left(G_{y}/G_{x}\right)$. The smoothing factor is adjusted for de-noising, and the edge points are disposed of by the lagging threshold. The true edge points are obtained after non-maximum suppression value treatment, and a thin edge line would be obtained in the form of a ridge, which prevents the occurrence of a double edge due to the second-order difference operator. In fact, the initial state of the FOD radar system is often located in a vertical direction to the airfield runway. Current knowledge indicates that the runway edges’ gradient angle is close to 90° or 0° in rectangular coordinates. When the airfield pavement is not clean, there could be some targets on the surface that cause unwanted edges inside. In this case, extra morphological corrosion and dilation processing would be utilized to eliminate the inner edge lines. Within the inspection range of the radar system, we can extract a minimum outer rectangle of the runway as a certain ROI area for further detection handling.

### 3.2. Spatial Domain Methods

**Figure 3:**
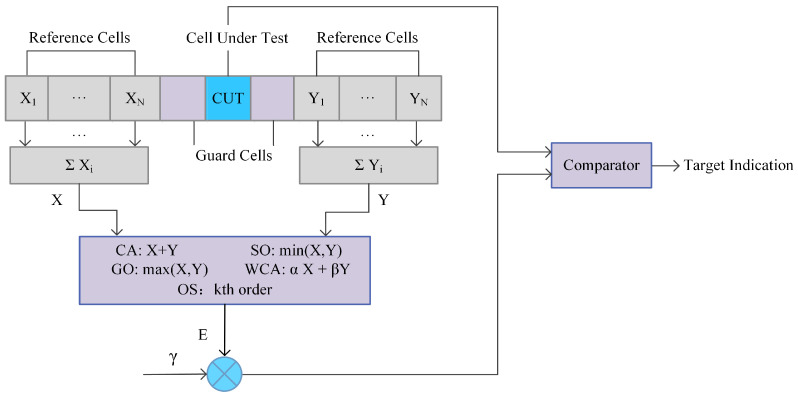
Common CFAR processors.

The constant false alarm rate (CFAR) algorithm is a typical auto-target detection method for the FOD system. Considering a cell average (CA) CFAR detector, the basic principle is expressed in Figure 3. The received beat frequency signal is operated by the FFT calculator and fed into the CFAR processor. Except for the guard cell, also called the cell under test (CUT), other reference cells participate in estimating ground clutter power. The detection threshold can be demonstrated as Equation (12). γ is the threshold multiplication coefficient that maintains the detection of false alarms.

(10) $$E=\frac{1}{2N}\sum_{i=1}^{N}\left(X_{i}+Y_{i}\right)$$

(11) $$\gamma =2N\left(P_{fa}^{-1/2N}-1\right)$$

(12) $$T=\gamma E=\gamma \frac{1}{2N}\sum_{i=1}^{N}\left(X_{i}+Y_{i}\right)$$

Meanwhile, the GO, SO, WCA, OS CFAR processors are calculated in a different estimation method by the front and the back reference cells. They all make use of spatial information. CA CFAR is effective under conditions of homogeneous or stationary interference. GO CFAR takes the maximum of ${\left\{X_{i},Y_{i}\right\}}_{N}$, which are also known as the leading and lagging parts. It works effectively in the clutter edges but results in detection loss when encountering multiple targets. SO CFAR utilizes the minimum part to estimate the noise power of ${\left\{X_{i},Y_{i}\right\}}_{N}$. It can resolve multiple target detection conditions but has difficultly in maintaining a constant false alarm rate in marginal clutter conditions. At the same time, WCA CFAR represents a compromise between GO and SO handling. OS CFAR chooses the kth cell as the detection threshold. It needs to sort the order first and is estimated without guard cells. It also has a certain ability to counter targets’ obscuration effects, although it could cause false alarms and loss of detection since it relies on the kth order.

### 3.3. Time-Domain Methods

As mentioned in Section 2, heavy background clutter is the primary interference for FOD detection. Moreover, researchers and our practical process have shown that traditional space-domain CFAR techniques such as CA are ineffective because the scattering characteristic of ground clutter varies drastically in the space domain. For actual applications, the scattering characteristics of a typical range of cells changes little in a certain period. Thus, the time-domain CFAR, i.e., clutter map (CM) processors, will achieve better performance.

**Figure 4:**
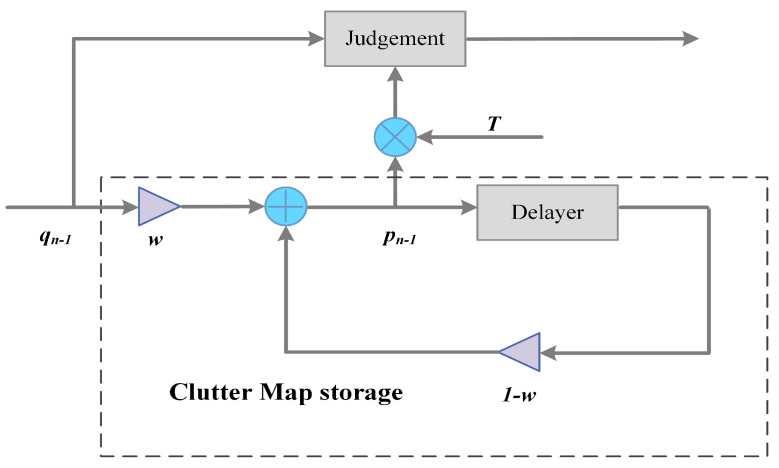
Clutter map processor diagram.

The clutter map CFAR processing method was initially proposed by Nitzberg [34] and approved forms were put forward later. It considers the relative stability of the radar clutter environment in the time domain. The main two steps are clutter map establishment and clutter map detection. A schematic diagram is shown in Figure 4.

The intrinsic property of the CM processor is a first-order autoregression model. Operating on the previous echoes information, it performs as an exponential weighted average process. The iteration function can be written as:

(13) $$p_{n}\left(k\right)=w\sum_{i=0}^{\infty }{\left(1-w\right)}^{i}q_{n-i}\left(k\right)$$

(14) $$\frac{q_{n}}{p_{n-1}}\begin{array}{c} \geq^{H_{1}}\\ {<}_{H_{o}} \end{array}T$$

The self-adaptation judge threshold is expressed as Equation (14). Combining the received signal, the clutter map could be established in the time sequence. To build a stable clutter map, the iteration number *L* and weight *w* need to make a trade-off. The greater the weight *w* is, the less time is needed to build up a stable clutter state, the less *L* is needed. Here, the steady state refers to a condition that satisfies the false alarm probability, and the detection performance is steady. The predefined threshold factor *T* is related to the maintained false alarm ratio. Rohling [35] defines an average detection threshold (ADT) to represent the performance of a detector. This means the average detection threshold when achieving the same detection probability under certain circumstances. The ADT of the clutter map technique is written as:

(15) $$ADT=T\sum_{i=1}^{L}w{\left(1-w\right)}^{i-1}=T\left[1-{\left(1-w\right)}^{L}\right]$$

The smaller the ADT, the better performance of the processor. Under a maintained false alarm ratio condition, a more extensive *w* would increase the CFAR loss, decrease the detection ratio, and need more time to reach a steady state. Therefore, in terms of limited scan background period *L* conditions, decision-makers should consider the advantages and disadvantages among iteration times and detection performance. The scan background period is equal to the iteration number defined above since iterative processing is performed with background data.

### 3.4. Explicit Definition of Quantitative Criterion

In order to evaluate the quantitative performance of the FOD surveillance radar, we adopt a confusion matrix expressed as Table 1. Detection is assumed as a binary result. The GT is the ground truth of the access data. Furthermore, the detection probability $P_{d}$, false alarm probability $P_{fa}$, probability of correct classification PCC, and Kappa coefficient are defined as Equations (16)–(21).

(16) $$P_{d}=\frac{TP}{TP+FP+FN}$$

(17) $$P_{fa}=\frac{FP}{TP+FP+FN}$$

(18) $$PCC=\frac{TP+TN}{N}$$

(19) $$KC=\frac{P_{o}-P_{e}}{1-P_{e}}$$

(20) $$P_{o}=PCC$$

(21) $$P_{e}=\frac{TP+FN}{N}\times \frac{TP+FP}{N}+\frac{TN+FP}{N}\times \frac{TN+FN}{N}$$

where N=TP+FP+FN+TN is sum of the whole sample of results, $P_{o}$ is the observation coincidence rate, which equals PCC, and $P_{e}$ is the expectation coincidence rate.

Compared to the common definition, i.e., $P_{d}$ is the ratio of true positive divided by true FOD targets, $P_{fa}$ is the exceeding true samples divided by true FOD targets. Our explicit quantitative criterion is more objective and reasonable. PCC contains all true positive and negative results that could reflect the constant false alarm ratio. $P_{d}$ and $P_{fa}$ are defined among TP, FP, FN, which ensure that the ratio does not surpass probability 1. Kappa coefficient KC is calculated by the detection coincidence rate and observation coincidence rate. It is used to evaluate the overall degree of similarity between the detection results and actual ground truth. It is more sensitive to the difference and indication performance of detectors as well. The closer the KC value is to 1, the more accurate the detection result that is measured. With this criterion, it is straightforward to depict the quantitative characteristic of one FOD detection system.

## 4. Experiments and Performance Evaluation

In this section, the runway ROI automatic extraction is applied to the complete algorithm. We compare our detection performance results to the spatial-domain CFAR algorithms with our defined quantitative characteristic criterion. In order to collect actual measured data, a surveillance FOD MMW radar system developed by AIRCAS was deployed at a civil airport in Beijing, China. The airfield runways consisted of asphalt pavement. The system description, operating mode, FOD targets, and the parameters of the MMW radar are given in Table 2. In particular, the carrier central frequency is $f_{c}$ = 93 GHz, and the duration of a single frequency sweep is $T_{r}$ = 1 ms with a coverage of more than 700 m. The transmitting power is 27 dBm, the bandwidth of sweep frequency is $B_{r}$ = 2 GHz, and the processing of the ROI area is in both polar and rectangular coordinates.

**Figure 5:**
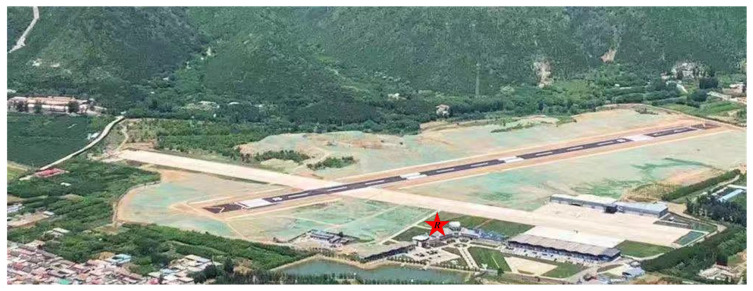
The actual experiment scene at a civil airport in China: an aerial map of the studied airfield, with the FOD millimeter-wave radar system’s location indicated by the letter *R*.

**Figure 6:**
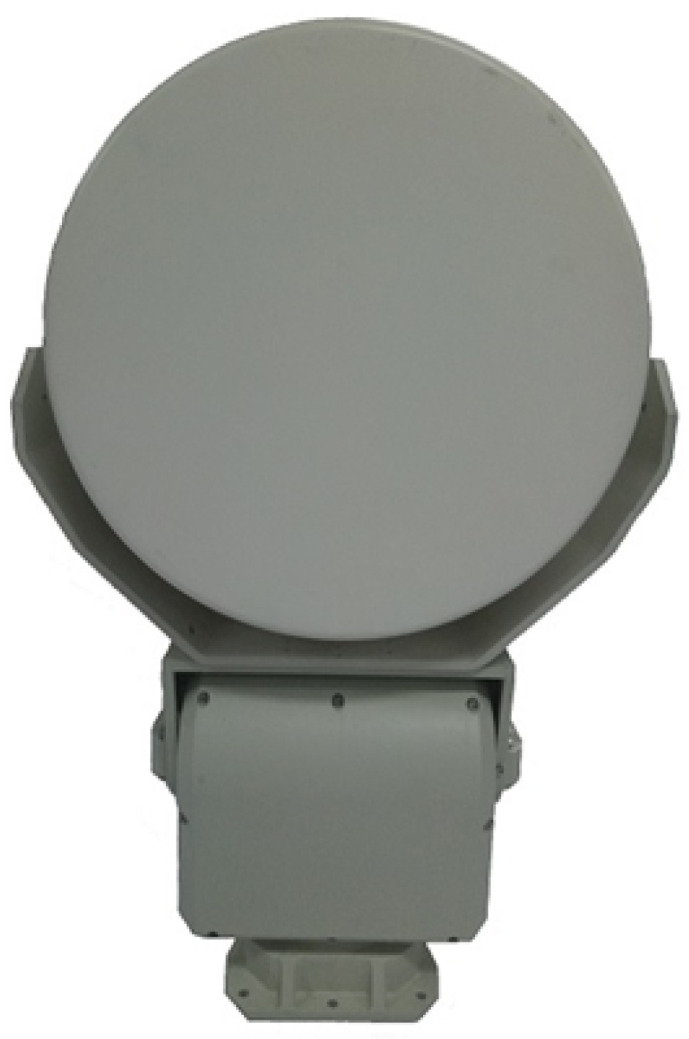
The proposed FOD millimeter-wave surveillance radar.

An optical map of the implement airfield location, obtained from Google Earth, is shown in Figure 5. Our designed FOD MMW radar was placed on a 9 m high platform on the top of the terminal building. Its running state is depicted in Figure 6. The system comprises a power module, servo module, radar module, and transmission lines. Rather than one-dimensional detection, with a short inspection runway range and strong metal target detection, the proposed method is focused on two-dimensional processing measurement, non-metallic targets such as golf balls, and an extensive runway monitoring range of over 660 m. Additionally, the region of interest runway pavement automatic extraction is applied after azimuth-range image processing. The acquired background data are used for clutter map establishment within several scanning circles. Then, the observation data are registered geometrically and detected by the clutter map CFAR. For the comparison of the detection performance, we perform a quantitative assessment using the explicit criterion that was previously defined (see Section 5). Next, we conduct an analysis of the proposed measurement method step by step.

Before collecting the data, the experimental scene was manually checked first to ensure that no FOD targets were on the pavement. The servo module drove the other modules in rotation. The millimeter-wave radar scanned the experimental scene and recorded the echo data of the ground clutter and observation scene. Since we apply a time-domain algorithm, the acquired background data would be used for clutter map establishment within some scanning cycles. The observation data would be measured for further treatment.

**Figure 7:**
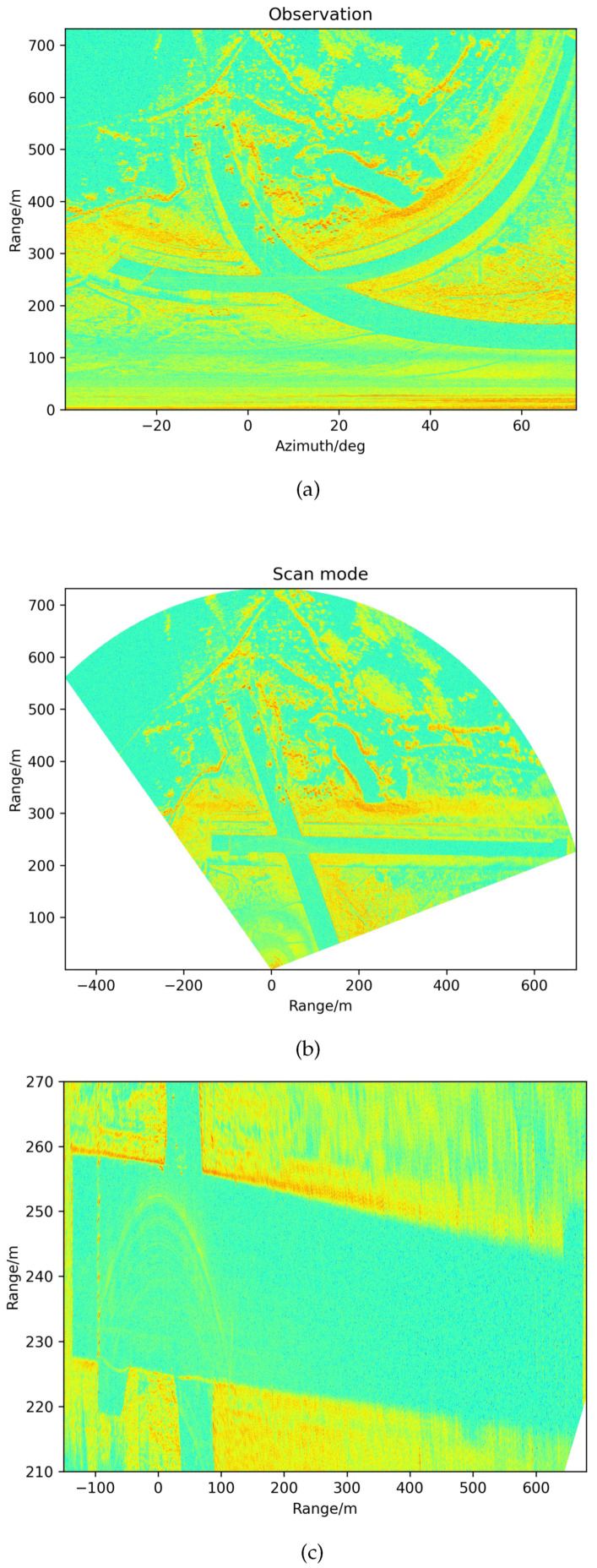
The azimuth range 2D imaging results. (**a**) Observation data in polar coordinates. (**b**) Observation data transformed to rectangular coordinates. (**c**) The runway region of interest area.

We first obtained the imaging results, as depicted in Figure 7. In particular, in Figure 7b, the scan mode is more visible to the human eye. Figure 7c shows the runway ROI area’s amplification effect from Figure 7b. There are two intersecting runways: the main inspection one is perpendicular to the initial state (azimuth scan: 0 degrees) of the FOD millimeter-wave radar. In Figure 7c, the point-like object on the left is the actual runway light and the arc-shaped band is the interference zone formed around the scene. Furthermore, the other one is not the inspection runway. It is clear that there are several aircraft parked on the latter sub-runway. As for background clutter map establishment, there is no need to clear everything on the pavement altogether. The existing runway lights can be established as a strong background with the clutter map and will not be detected as targets in time series. In this experiment, we measured ten scanning circles in the form of background data for clutter map establishment. Every piece of 2D imaging data was processed with ROI extraction. During updating of the clutter map measurement, the ROI area of a fixed bounding box can be set, which can then be used for the detection of the registered observation data.

**Figure 8:**
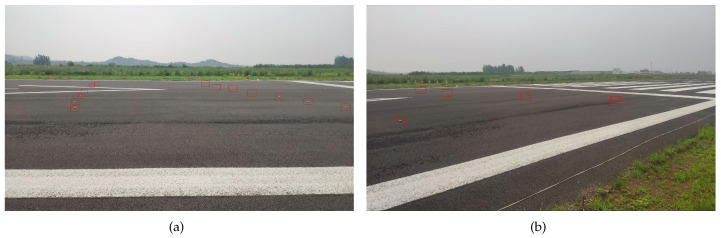
Location of the golf balls on the pavement. Since the targets are too small in the image, they are highlighted in red rectangular boxes.

In order to validate the proposed method with non-metallic targets and in a long-distance situation, the optical placement images are expressed in Figure 8 through two different viewing angles. We used ordinary golf balls as detection objects, which are highlighted in red rectangular boxes. The diameter of the golf balls was 43 mm, and the radar cross-section was less than −28 dBsm. They were placed at the far-end of the pavement. The airfield was slightly wet because of the drizzling weather. Due to the tiny targets and the long distance of the runway, it was not easy to determine the targets’ location in the ROI figure.

**Figure 9:**
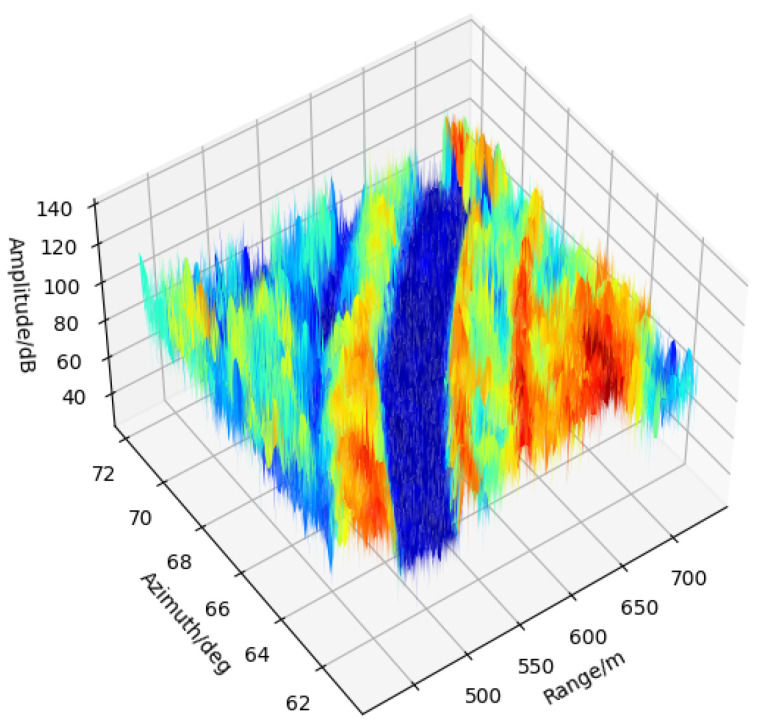
The establishment of background clutter map in 3D view.

We used ten scanning circles as background data to establish the time-iterative clutter map. A view of a three-dimensional graph is shown in Figure 9. While we focus on a long distance over the 500 m range, the runway’s amplitude is relatively lower than that of grassland and other objects. The intensity of the runway pavement higlighted in blue also shows absolute stability since the clutter map is iterated of ten calculations. In the next detection process, the clutter map updating takes place.

**Figure 10:**
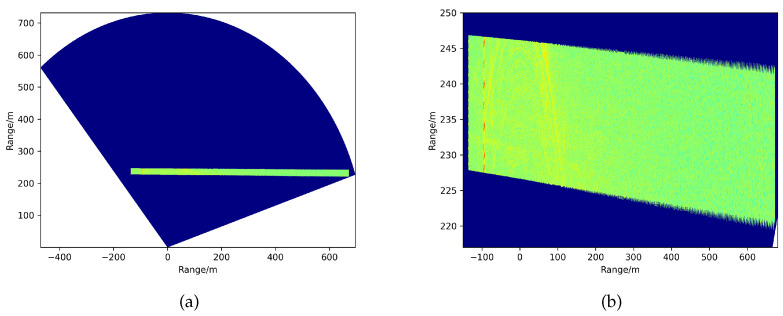
The runway ROI extraction results. (**a**) The extracted main inspection runway in the whole range. (**b**) The local amplification of extracted runway.

Based on the bilateral filtering, edge strength, and edge direction, we could acquire the main inspection runway expressed in Figure 10. Here, we have the square boundary frame in the whole range, as Figure 10a shows. A illustrated by the geometry in Figure 1, the azimuth scanning angle is not beamwidth, but the angle is formed with a fixed initial direction. The direction of runway edges is approximately parallel or perpendicular to the radar zero scanning angle. The radar system is located in the X-axis zero position. The amplification of the runway ROI extracted from the clutter map is complete and ’clean’ in Figure 10b. The strip lights on the left of the runway pavement are considered as inherent background clutter. It can be observed that the directions of the edges are not strictly perpendicular to each other. The reason for this is associated with the radar’s initial installation position. There is a certain amount of redundancy when using edge direction information to minimize the lack of boundaries.

**Figure 11:**
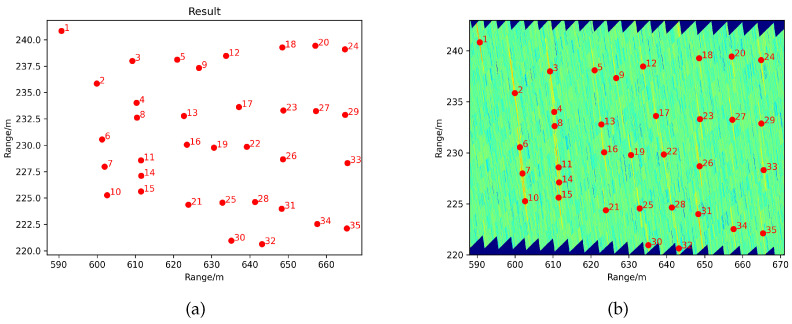
The detection results. (**a**) Taking one centroid point for each object. (**b**) The detection points together with amplification of local runway ROI.

The azimuth resolution becomes 5 m at a long detection distance, and the resolution range remains the same. The detection results are given in Figure 11. The results are converted to the Cartesian coordinate system. In order to display the detection results of the ROI area more clearly, we also show the detection results on the ROI radar image in Figure 11b. While the targets occupy smaller cells compared to the resolution cells, the targets perform as banded pixels, as Figure 11b shows. Though the detected targets take up a specific area in the intensity graph, we selected the centroid of the identical object as the final position feedback that is depicted in Figure 11a. It can be seen that nearly all the golf balls were identified correctly within the system resolution. The minimum interval of detectable target placement was less than 5 m. The processing indicators show that our designed FOD millimeter-wave radar system is superior to the requirements outlined by the FAA [5].

For further quantitative assessment, we adopt the defined criterion (see Section 5) to evaluate the performance of the FOD surveillance radar. Compared with the CA, GO, and SO CFAR algorithms, the experimental measurement criterion is expressed in Table 3. Since the former three algorithms are spatial-domain processors, the processing parameters are set to be the same: $P_{fa}=10^{-6}$, ten guard cells, and 16 reference cells. Among them, the GO obtained a larger value of both $P_{d}$ and $P_{fa}$ than CA. The SO detected the most placed objects TP, but there existed many false alarms regarding objects FP on the runway edge, which led to a low $P_{d}$. Overall, the Kappa coefficient KC indicator shows the general performance of the detectors. According to the detection rate $P_{d}$, the false alarm rate $P_{fa}$ of the golf balls at different ranges from 590 m to 670 m, and KC, the detection rate of the CM CFAR algorithm is better, and the false alarm rate is lower as well. The PCC values are more relevant to the setting parameters $P_{fa}$. Although its value is closer to 1, the reference value is not too large. Therefore, there is no need to classify the false alarm and actual targets to achieve better performance, because the target occupies few pixel units in long-range conditions, which means that the use of statistical information to extract features will no longer work. It is also verified that a typical range cell’s scattering characteristics change little in a certain period for actual applications. This criterion result is consistent with our previous analysis in Section 3.3.

## 5. Discussion

In this study, we investigated the current research on the FOD MMW radar system and target detection method of common debris at airports. We found that there are shortcomings in existing FOD radar systems and detection algorithms, i.e., the lack of remote object detection and low processing efficiency. Consequently, we proposed an automatic target detection method for small FOD objects in a long inspection range and defined the explicit performance criterion. Inspired by the confusion matrix for image classification, we provided a new quantitative index for evaluating performance. It could be utilized as a new and explicit criterion suited to FOD target detection evaluation.

The experimental results in Section 4 manifest the effectiveness of our method. The comparison experiment was performed under the same detection parameters. Concerning the spatial domain, algorithms employ the same guard cells, reference cells, and threshold set $P_{fa}=10^{-6}$, while the CM processor does not need guard or reference cells and the threshold is set to the same $P_{fa}$ for comparison. Compared with the other algorithms, this paper’s time-domain processor has advantages in terms of detection probability, detection false alarm probability, and overall performance coincidence rate between ground truth and detection results. As for the small targets, the experimental targets are standard objects in accordance with FAA [5]. The proposed method could still obtain robust results when other objects are greater than or equal to the RCS of the experimental object.

The proposed approach is robust with regard to climate conditions. The used experimental data were collected under drizzling rain. Figure 8 shows the wet surface on the pavement. From the perspective of actual application, the runway has a structure with a certain slope, meaning that the central line is high and the two sides are low (see Figure 1). This sloped structure could help water to flow to the grassland on both sides, so it would not come into contact with stagnant water. However, snow conditions would cover the pavement, reduce the friction coefficient of the runway surface, and prevent the aircraft from taking off. Under foreseeable circumstances, snow coverage of the target will hinder the detection process, especially small-sized targets. From the perspective of the radar signal, water and snow in the air would cause a decay in signal amplitude. Referring to the Radar Handbook, the millimeter-wave band signal attenuation caused by rain or snow is less than 2 dB in moderate rain and snow conditions within a 1 km range. The impact for the target amplitude and detection process is limited. As for the clutter map establishment, the pavement would merely be a thin layer of water on the surface. The processed clutter map will automatically update in the time domain. Our measured rainy data could validate the robustness of the method while the snowy measurement is analyzed.

To conclude, we achieved the automatic target detection of remote and small debris on airfield pavement for an FOD surveillance radar. It comprises runway ROI extraction based on signal form analysis, background clutter map establishment in the time domain, observation data registration, and clutter map detection processing. The experiment was carried out in an actual civil airfield runway in Beijing, China. Here, we measured the method’s effectiveness, and the designed FOD MMW surveillance radar has an inspection radius covering 660 m of the runway distance. Rather than qualitative evaluation of the results, we assessed the performance with our clearly defined evaluation indicators. The experimental results demonstrate that our method performed well in the remote inspection and non-metallic debris detection scenario with automatic processing. In the near scenes and for large-scale debris objects, it was showed excellent detection performance. At the same time, this method does not require manual data segmentation processing or prior information of the runway position. The high automation level and high working efficiency make it more competitive in real-time applications. It also offers processing support for the fusion with information acquired through radar and other sensors such as optical–electronic equipment and further decisions by airfield personnel.

## 6. Conclusions

In this paper, we proposed the complete automatic target detection of small debris in a long-distance scenario and a quantitative criterion for an FOD surveillance radar. We found that there are shortcomings in some existing systems and detection algorithms among the current FOD surveillance radar system methods, i.e., the lack of remote object detection and the low processing efficiency. In this regard, we proposed an automatic target detection method for small FOD objects at a long inspection distance and defined the explicit performance criterion. In accordance with FMCW signal form and clutter modeling of the airfield scenes, a runway ROI extraction algorithm is employed to concentrate on the meaningful areas, which could improve the efficiency. Through time-domain processors, the clutter map CFAR detection algorithm performed better in terms of detection probability, false alarm probability, and airfield scenario robustness. Experimental data were acquired to verify its superiority at a civil airport in Beijing, China. Compared to standard spatial CFAR algorithms, the results demonstrate that the proposed automatic detection method can provide precise criterion indicators with small targets at long inspection distances covering up to 660 m of the runway. It also has excellent detection performance in near scenes and higher target RCS than golf balls since this is more helpful for object detection. In the future, we will continue to improve our method so that it can be applied to a more complicated pavement environment. Moreover, we will attempt to apply some super-resolution techniques to overcome the limitations of azimuth resolution or increase the FOD radar system’s complexity. Other target classification approaches will also be taken into consideration as supplementary approaches.

## Figures and Tables

**Table 1:** Definition of quantitative criterion.

Performance	Detect Changed (P)	Detect Non-Change (N)
GT Changed (T)	TP	FN
GT Non-changed (F)	FP	TN

**Table 2:** AIRCAS designed FOD radar parameters.

Parameter	Value, Description
Radar Waveform	FMCW
Central Frequency	93 GHz
Transmit Power Level	27 dBm
Antenna Gain, Polarization	38 dBi, H Polarization
Azimuth/Elevation Beam Width	0.6°/4°
Range Resolution	0.07 m
Cross-range Resolution	5 m (maximum range)
Minimum Detectable RCS	−28 dBsm
Detection Range	700 m
Runway Width	30 m
Runway Length	660 m
Radar Deployment	9 m above the ground and 120 m away from the runway
Scan Period	less than 1 min
Detect FOD Samples	Described by FAA advisory circular [5]

**Table 3:** Performance with quantitative criterion.

Performance	$P_{d}$ (%)	$P_{\mathrm{fa}}$ (%)	PCC (%)	KC
CA CFAR	73.68	2.63	99.9573	0.85
GO CFAR	76.92	5.13	99.9554	0.87
SO CFAR	57.14	41.27	99.9361	0.73
CM CFAR	94.59	0	99.9989	0.97

## Data Availability

The data presented in this study are available on request from the corresponding author. The data are not publicly available due to privacy.

## Abbreviations

The following abbreviations are used in this manuscript:

ROI	Region of interest
TP	True positive
FP	False positive
TN	True negative
FN	False negative
AIRCAS	Aerospace Information Research Institute, Chinese Academy of Sciences.
